# A mixed-methods study of multi-level factors influencing mammography overuse among an older ethnically diverse screening population: implications for de-implementation

**DOI:** 10.1186/s43058-021-00217-7

**Published:** 2021-09-26

**Authors:** Jessica D. Austin, Parisa Tehranifar, Carmen B. Rodriguez, Laura Brotzman, Mariangela Agovino, Danya Ziazadeh, Nathalie Moise, Rachel C. Shelton

**Affiliations:** 1 Department of Epidemiology, Columbia University Mailman School of Public Health, New York, NY USA; 2 Department of Sociomedical Sciences, Columbia University Mailman School of Public Health, 722 W 168th Street, New York, NY 10032 USA; 3 Herbert Irving Comprehensive Cancer Center, Columbia University Medical Center, New York, NY USA; 4 Department of Medicine, Columbia University Irving Medical Center, New York, NY USA

**Keywords:** De-implementation, Overuse, Mammography screening, Mixed-methods, Multi-level

## Abstract

**Background:**

There is growing concern that routine mammography screening is overused among older women. Successful and equitable de-implementation of mammography will require a multi-level understanding of the factors contributing to mammography overuse.

**Methods:**

This explanatory, sequential, mixed-methods study collected survey data (*n*= 52, 73.1% Hispanic, 73.1% Spanish-speaking) from women ≥70 years of age at the time of screening mammography, followed by semi-structured interviews with a subset of older women completing the survey (*n*=19, 63.2% Hispanic, 63.2% Spanish-speaking) and providers (*n*=5, 4 primary care, 1 obstetrics and gynecology) to better understand multi-level factors influencing mammography overuse and inform potential de-implementation strategies. We conducted a descriptive analysis of survey data and content analysis of qualitative interview data. Survey and interview data were examined separately, compared, integrated, and organized according to Norton and Chambers *Continuum of Factors Influencing De-Implementation Process.*

**Results:**

Survey findings show that 87.2% of older women believe it is important to plan for an annual mammogram, 80.8% received a provider recommendation, and 78.9% received a reminder in the last 12 months to schedule a mammogram. Per interviews with older women, the majority were unaware of or did not perceive to have experienced overuse and intended to continue mammography screening. Findings from interviews with older women and providers suggest that there are multiple opportunities for older women to obtain a mammogram. Per provider interviews, almost all reported that reducing overuse was not viewed as a priority by the system or other providers. Providers also discussed that variation in mammography screening practices across providers, fear of malpractice, and monetary incentives may contribute to overscreening. Providers identified potential strategies to reduce overscreening including patient and provider education around harms of screening, leveraging the electronic health record to identify women who may receive less health benefit from screening, customizing system-generated reminder letters, and organizing workgroups to develop standard processes of care around mammography screening.

**Conclusions:**

Multi-level factors contributing to mammography overuse are dynamic, interconnected, and reinforced. To ensure equitable de-implementation, there is a need for more refined and empirical testing of theories, models, and frameworks for de-implementation with a strong patient-level component that considers the interplay between multilevel factors and the larger care delivery process.

**Supplementary Information:**

The online version contains supplementary material available at 10.1186/s43058-021-00217-7.

Contributions to the literature

- Routine mammography screening is widely implemented but offers limited health benefits while posing unnecessary health risks for older women with diminishing life expectancy and competing health risks.
- De-implementation of routine mammography screening in older women offers an opportunity for advancing the science of de-implementation.
- This study provides a grounded empirical example illustrating how factors at one level (patient) can be perpetuated or reinforced by factors across multiple levels of influence (interpersonal, provider, system).
- Considering the experiences and views of populations underrepresented in de-implementation science research, as well as the larger process of care delivery is important in developing and implementing multi-level de-implementation strategies.

## Introduction

In recent years, there has been a growing emphasis on de-implementing the delivery of unnecessary or low-value healthcare [1–5]. De-implementation refers to reducing, discontinuing, or replacing healthcare that is ineffective, inappropriate, and/or unproven. This area has received growing attention among funders and healthcare systems given that it is a vital part of improving overall population health, increasing the quality of care, decreasing unnecessary costs, and minimizing patient harm [1]. Several campaigns, such as “Choosing Wisely [6],” have focused on efforts to decrease healthcare overuse for a range of health issues (e.g., prostate cancer screening, back pain imaging, cardiac and vitamin D screening, and prescribing opioids for migraine) [4, 7, 8]. Overuse of care and services in the context of cancer—including overscreening, overdiagnosis, and overtreatment—is common and can cause unnecessary harm, emotional distress, and increased cost for patients and the healthcare system [8–10]; however, to date, de-implementation in the context of routine mammography screening for older adults has not been thoroughly examined and offers an excellent opportunity for advancing the science of de-implementation in clinical and healthcare contexts [1, 2, 11].

There is growing concern that mammography screening is overused in older women in the USA, offering limited benefits while posing unnecessary health risks [12]. Mammography screening is associated with a 15 to 25% reduction in breast cancer mortality after 10–15 years [13], but evidence suggests that it does not lead to a significant reduction in breast cancer mortality among women with limited life expectancies and greater competing health risks [14–17]. Rather, it may pose substantial and immediate harms, such as anxiety, financial, and time burden, as well as diagnosis and treatment of tumors that would not have resulted in death [15, 18–20]. Current professional guidelines in the USA (e.g., US Preventive Services Task Force, American Cancer Society, American College of Physicians) do not support routine mammography screening for women ages 75 and above [21–24]; yet, 56% of women 75 years and older report a recent mammogram, half of whom have a life expectancy less than 10 years [25]. In the USA context, lack of a national mammography screening program and inconsistencies across professional organizations around how best to approach screening for older women likely contribute to continued use of routine mammography in older women.

While prior studies have explored factors promoting mammography utilization and adherence, factors contributing to mammography overuse among older women in the USA remains limited and largely unknown [26–28]. A recent narrative review of older women’s perspectives around mammography screening found that older women are largely uninformed about cancer screening guidelines and the potential harms of mammography screening [29]. Other studies also report that most healthcare providers do not discuss the uncertain balance of mammography benefits and harms, and over 70% recommend mammography screening to their older patients [30–32]. Changes to guideline recommendations over time also create inconsistencies, confusion, and mistrust that can further hinder de-implementation efforts [33]. Further, healthcare systems may be less likely to reduce or discontinue screening efforts if it generates considerable revenue [1]. Thus, successful de-implementation of mammography screening in older women poses a substantial and complex challenge that requires an understanding of multi-level factors contributing to mammography overuse across different contexts.

Understanding the de-implementation of routine mammography screening in older women offers the opportunity for advancing the science of de-implementation in clinical and healthcare contexts. Among the most critical next steps is ensuring that de-implementation efforts are equitable [33–35]. To date, few studies have included the perspectives of older, racial/ethnic minority women around mammography screening [29]. Understanding the perspectives and experiences of these populations is critical for ensuring de-implementation efforts do not further exacerbate existing cancer health disparities [34, 36]. To this end, the purpose of this pilot study was to explore multi-level factors contributing to mammography screening overuse at one of the largest ambulatory care settings in New York City and to elicit potential de-implementation strategies to reduce mammography screening overuse at the patient, provider, and system levels.

## Methods

This explanatory-sequential mixed-methods pilot study collected in-person survey data from older women followed by in-depth interviews with a subset of older women and providers serving as key informants to better understand factors influencing mammography overuse at the patient, provider, and system levels. The survey and interview design were informed by an extensive review of the literature on prevalence, benefits, harms, attitudes, and practices associated with breast cancer screening in older women, and national recommendations for mammography screening. The survey among older women was designed to provide a broad understanding of potential factors contributing to mammography overuse, while the interviews among older women were designed to add depth and context to survey findings and to elicit insights into the processes and mechanisms through which routine mammography is reinforced. The provider interviews were designed to corroborate and inform findings from older women interviews and explore potential de-implementation strategies. The study was approved by the Columbia University Medical Center Institutional Review Board. All participants provided written and/or verbal consent at the time of recruitment, survey, and interviews. Older women received separate gift cards for completing the survey and interview. Providers did not receive compensation for participation.

### Setting and sample

To sample older women who were actively undergoing mammography screening, we recruited women over the age of 70 years, with no history of breast cancer, as they presented for their screening mammography appointment at a clinic in New York City (~14,000 mammograms/year) to complete an in-person survey. The catchment area the mammography screening clinic serves includes a large proportion of Hispanic, Spanish-speaking women (>75%) with high rates of routine mammography screening (87.9%). While guideline recommendations are for women 75+, we included women 70 to 74 years to explore their perspectives and experiences around mammography screening decision-making as they approach the age for which guidelines for screening change. All women who completed the in-person surveys agreed to be contacted for follow-up studies and received a mailed invitation to participate in semi-structured interviews; a subset subsequently completed the semi-structured interviews by phone or in-person. Interviews continued until no new ideas or information emerged, deeming the data as saturated [37].

After collecting survey and interview data from older women, we recruited a purposeful sample of providers to serve as key informants to corroborate and expand upon findings from older women surveys and interviews. Initially, we identified primary care providers by email and in-person who provide care to older women in our healthcare system and have experiential knowledge and insights into mammography screening/referral processes. Based on initial findings from primary care providers, our team aimed to expand its provider reached to include the perspectives of obstetrics and gynecology (OBGYN) providers who are also involved in mammography screening decisions among older women; one completed a semi-structured interview.

### Data collection

Table 1 provides a detailed summary of the study methods including the data collection source, domain assessed, and example survey and interviews items. Between January and July of 2018, a total of 52 older women were approached by a bilingual member of the research team during a mammography screening clinic appointment to participate in an in-person survey. A member of the research team reviewed the mammography clinic schedule to identify eligible participants on select days. All 52 older women agreed and completed the in-person survey (27% in English and 73% in Spanish). The survey collected sociodemographic, clinical, and mammography screening data to assess older women’s knowledge, attitudes, and beliefs (motivation, perceived barriers, perceived seriousness, perceived severity) around mammography screening, screening communication, and shared decision-making. Between June and August 2019, two members (1 bilingual English and Spanish; 1 English-speaking) of the research team trained in qualitative methods completed 19 semi-structured interviews with a sub-sample of older women that completed the survey. The interview guide elicited older women’s attitudes and beliefs around mammography screening, communication and shared-decision making, the process of obtaining a mammogram, and perceptions of and sources of healthcare overuse more broadly. Characteristics of the survey and interview population of older women are provided in the supplemental materials. Next, we conducted 5 provider semi-structured interviews (4 primary care providers; 1 OBGYN) between October 2019 and February 2020. The primary goal of the provider interviews was to corroborate findings from older women surveys and interviews and to gain additional insight into why older women continue to get screened. The provider interview guide elicited the provider’s knowledge, attitudes, and beliefs towards mammography screening guidelines, how they decide and communicate with older women about the need for mammography screening, and the referral process. Additionally, we asked providers their perceptions of sources of mammography overuse and potential strategies to reduce overuse.

**Table 1:** Summary of data collection methods and domains

Domain	Data source	Ex. item(s)
Knowledge of mammography screening guidelines	Patient surveys	How often do you think you need to get a mammogram?
	Provider interview	What guidelines or clinical recommendations do you follow when recommending for or against mammography screening?
Attitudes/beliefs towards mammography screening	Patient survey	If I get a mammogram and nothing is found, I do not worry as much about breast cancer. It is important for me to plan to have a yearly mammogram.
	Patient interview	Can you tell me why you get mammograms? In general, how would you describe your experiences of getting mammograms?
	Provider interview	What are your perceptions of the utility of (breast cancer screening) guidelines in clinical practice? How do you feel about recommending mammography screening for women in their 70s? 80s?
Communication around mammography screening	Patient survey	Have you ever had a conversation with a doctor/family/friend about whether you should stop screening mammography?
	Provider interview	Tell me about how/whether you discuss mammogram screening with your patients?
Shared decision-making	Patient survey	When you have to make a decision about your health, how often do you consult family, friends, neighbors, or caregivers? Please tell us if you strongly agree, agree, disagree, or strongly disagree with the following statements. My doctor/family members decide when I should be screened for health problems.
	Patient interviews	How do you make the decision to get a mammogram?
	Provider interviews	What factors influence whether you discuss/refer for mammography screening among your patients?
Mammography screening process	Patient survey	Within the past 12 months, has a doctor or other healthcare provider recommended that you have a mammogram? Within the past 12 months, have you received a letter, phone call, or email reminding you to make an appointment for a mammogram?
	Patient interviews	Can you take me through the process of how you came to get your mammogram?
	Provider interviews	Tell me about the process you use for referring/recommending patients for mammograms either in the patient/provider appointment or at a system level (e.g., in-person, letter, other)?
Healthcare/screening overuse	Patient interviews	Have you ever heard or thought about the issue of getting too much healthcare? Please tell me about your thoughts. In your opinion, what are some of the reasons or motivations for doctors to order too many screening tests or medical services? Are there other reasons that you think contribute to patients receiving too much screening or medical services?
	Provider interviews	What are your perceptions of potential over-screening or over-use of mammography among certain groups?
Potential de-implementation strategies	Provider interviews	What are ways that providers or systems could better support patients in being adherent to mammography screening guidelines?

### Data analysis

Researchers trained in a mixed-methods analysis performed a descriptive analysis of survey data and thematic analysis of interview data focusing on factors influencing mammography overuse at the patient, provider, and system levels. We analyzed and organized findings according to Norton & Chambers framework (*Continuum of Factors Influencing De-implementation Process)* [2] to facilitate a deeper understanding of factors contributing to mammography overuse at the patient, provider, and system levels (e.g., patient and provider attitudes and beliefs, norms, system leadership). This organizing framework recognizes that factors influencing de-implementation efforts operate at multiple socioecological levels (e.g., patient’s beliefs and trust, provider attitudes and self-efficacy, system-level leadership, and societal or cultural norms). The framework also calls for multi-level strategies (e.g., techniques, approaches, tactics, or methods) that align with and address pre-identified factors within a specific context and population. While this framework has not yet been empirically applied to understand mammography overuse, it is intended to serve as a starting point that can be refined as it is applied to different populations and settings, including historically marginalized groups.

The survey and interview data were analyzed separately, compared, and then integrated during the data analysis phase. Descriptive statistics of survey demographics and key variables were calculated. Two members (JA and LB or JA and DZ) of the research team performed exploratory thematic content analysis using a deductive-inductive approach on all interview transcripts [38]. Each transcript was first reviewed in its entirety to allow themes and domains to emerge. For the older women interview data, a deductive code structure based on initial survey findings was applied to half of the transcripts during an initial open coding session. As new ideas emerged, the research team considered inductive themes, allowing the codebook to evolve [39, 40]. The revised codebook was then applied to all transcripts. The team coded the transcripts independently before coming together to compare codes. All coding discrepancies were resolved through consensus and analysis continued until no new ideas emerged eluding to saturation [37]. The same coding process was followed for the provider interviews using data from both older women surveys and interviews to inform the codebook structure. Next, key findings from each data source was presented, discussed, and agreed upon by the research team (JA, RS, PT, NM). We combined and triangulated quantitative and qualitative data from all sources and organized data according to Norton & Chambers framework [2] to identify factors at the patient, provider, and system-level contributing to mammography overuse. We also grouped potential de-implementation strategies proposed in provider interviews by the level of influence (e.g., patient, provider, system). Survey data is presented first, followed by qualitative interview data for older women and providers. Quantitative survey data was analyzed using SAS 9.4 [41] and qualitative data was organized and analyzed using NVivo software [42].

## Results

### Older women survey findings

Older women completing the patient survey were between the ages of 70 to 89 (mean 74.6) and representative of the population served by the mammography screening clinic. The majority of participants were Hispanic (73.1%), Spanish-speaking (73.1%), and 80.8% were born outside the USA (61.5% Dominican Republic). In addition, 75% had less than a college degree with marginal (28.9%) or low health literacy (28.9%). Approximately 83% reported having three or more self-reported doctor-diagnosed chronic conditions and only 26.9% perceived their health to be very good or excellent. Finally, 13.5% of older women reported a family history of breast cancer and 44.2% reported ever receiving a call back for additional diagnostic tests after a routine mammogram.

Table 2 provides a summary of key survey findings. Women have been receiving mammograms, on average, for 30.7 years (SD 8.1) and in terms of mammography frequency, 63.5% thought women should receive a mammogram once a year. In addition, 68.0% of older women perceived that getting a mammogram meant that they did not have to worry about breast cancer, 71.4% believed the treatment would not be as bad, and 87.8% believed that their chances of dying from breast cancer would decrease by getting a mammogram. All but one older woman reported that they “agree” or “strongly agree” that having a mammogram will help find breast lumps early (98.0%). Despite 79.6% of older women reporting that they “rarely” or “never” worry about breast cancer, 87.2% of women “agree” or “strongly agree” that it is important to have an annual mammogram.

**Table 2:** Survey items on older women’s perspectives around mammography screening and decision-making (*n*= 52)

	***n*** (%)
Perceived mammogram frequency	
Once a year	33 (63.5)
Every 2 years	6 (11.5)
Every 3 years/as needed/other	13 (25.0)
Years of screening (mean, SD)	30.7 (8.1)
If I get a mammogram and nothing is found, I do not worry as much about breast cancer.	
Agree or strongly agree	34 (68.0)
Disagree or strongly disagree	16 (32.0)
Having a mammogram will help me find breast lumps early.	
Agree or strongly agree	49 (98.0) 1 (2.0)
Disagree or strongly disagree	
If I find a lump through a mammogram, my treatment for breast cancer may not be as bad.	
Agree or strongly agree	35 (71.4)
Disagree or strongly disagree	14 (28.6)
Having a mammogram will decrease my chances of dying from breast cancer.	
Agree or strongly agree	43 (87.8)
Disagree or strongly disagree	6 (12.2)
Worry about breast cancer	
Rarely or never	39 (79.6)
Sometimes	7 (14.3)
Often/all the time	3 (6.1)
Perceived breast cancer seriousness^*^	8.5 (2.3)
Important to have a yearly mammogram.	
Agree or strongly agree	41 (87.2)
Disagree or strongly disagree	6 (12.8)
Provider recommended mammogram	
Yes	42 (80.8)
No	9 (5.8)
Ever told by the provider to stop mammograms	
Yes	7 (13.5)
No	45 (86.5)
Doctor decides when I should get screened for health problems.	
Agree	42 (80.8)
Disagree	5 (9.6)
Spoke with family about stopping mammograms	
Yes	6 (11.5)
No	46 (88.5)
Spoke with a friend about stopping mammograms	
Yes	6 (11.8)
No	45 (88.2)
Family members decide when I should be screened for health problems.	
Agree	10 (19.2)
Disagree	37 (71.2)
Number of family members that consult before making health decisions at least once in a while.	
None	10 (19.2)
One	11 (21.2)
Two	15 (28.8)
Three	7 (13.5)
Four or more	9 (17.3)
Consult a close friend, neighbor, or caregiver before making health decisions	
None	32 (61.5)
One	16 (30.8)
2 or more	4 (7.7)
Received a reminder in the past year (e.g., letter, call, email)	
Yes	41 (78.8)
No	11 (21.2)

Notes: ^*^Scale is between 1 and 10, where 1 is “not serious” and 10 is “extremely serious”

Over 80% of older women reported that their provider continues to recommend mammograms, and 86.5% indicated that they have not discussed stopping mammograms with any of their providers. Among those that did have a conversation with their provider to stop mammography screening (*n*=7), the majority were Hispanic (71.4%), foreign-born (71.4%), reporting fair to poor health (71.4%), and no history family history of breast cancer (100%). Only 11.8% of older women discussed stopping mammograms with a family member and/or a friend. Further, nearly 80% reported receiving a letter, a phone call, or email in the last 12 months reminding them to make an appointment for a mammogram. More generally, when making decisions about their health, 89.4% of older women “agree” that their provider decides when they should get screened for health problems, 80% consult at least one family member when making health decisions, and 38.4% consult a friend, neighbor, or caregiver.

### Older women interview findings

We conducted semi-structured interviews with a subset of older women who completed the survey to elicit their attitudes and beliefs towards mammography screening to provide depth and context to survey findings. There were no significant differences in sample characteristics between the interview or survey population (see Supplement). Interview findings are presented by key themes: (1) older women intend to and are encouraged to receive an annual mammogram, (2) there are many opportunities for older women to obtain a mammogram, and (3) older women are unaware of or did not perceive that they experienced healthcare overuse.

#### Older women intend to and are encouraged to receive an annual mammogram

We found that the majority of older women believed that getting a yearly mammogram was important to stay healthy and for detecting and treating breast cancer at an early stage. These beliefs were often shaped or encouraged by personal experiences with mammography screening (e.g., receiving a call to return for additional diagnostic tests), receiving a provider recommendation or reminder letter, and/or by knowing someone diagnosed with or dying from breast cancer. In turn, many women said that they intended on continuing mammogram screening. As a 71-year-old, Hispanic woman summarized:

I’m very protective of my appointments and my specialists, because one never knows, by avoiding medical attention, when one can develop a problem that you wouldn’t even know about, or feel symptoms for, and it could be serious. So I’m very protective of my appointments, I get my mammogram every year.

#### There are many opportunities to obtain a mammogram

We asked older women to describe the process of how they came to get a mammogram. In most cases, women said that their provider would recommend and/or refer them to get a yearly mammogram. Several women also stated that they received a reminder letter or a phone call reminding them to get a yearly mammogram. This reminder would sometimes prompt them to call their provider’s office for a referral or call the mammography screening clinic directly to obtain an appointment. A few women also stated that the letters served as a personal reminder to discuss mammography screening with their providers at their yearly appointment. As a 72-year-old, Hispanic woman described:

Well, my primary care doctor reminds me and they also send me the reminder, and if they don’t remind me, I see in the paper [letter] I have that it’s time to get it.

#### Older women are unaware of or have not experienced healthcare overuse

We described the concept of overuse/unnecessary healthcare to gather information on older women’s understanding and opinions/views. We asked women if they ever heard of or experienced “unnecessary” or “excessive healthcare,” herein referred to as overuse, and if they ever received excessive or unnecessary care. The majority of participants had not heard of or experienced healthcare overuse and many reinforced the importance and necessity of routine medical care, including mammograms, for detecting illness. As a 71-year-old, Hispanic woman reported:

I think the doctor tells you the care you need, at the moment you need it ... I don't think it's excessive, because, if you are going to have a mammogram, and the mammogram does not work out well, he’ll refer you to the professional.

We also asked women reasons for overuse more broadly and specific to mammography screening. The majority of women reiterated that all care is important but several reports that providers may give care that is not necessary if it is covered by insurance. Older women also identified a number of other potential reasons for healthcare overuse including patient requests for unnecessary care, patient non-adherence, perceptions that more care is better, provider fear of missing a diagnosis, and healthcare system fragmentation. As one 78-year-old, Hispanic woman described:

There are people who think that it’s [overuse/unnecessary or excessive care] to collect insurance, either insurance, or for the person, that’s the reason they do it. I think that maybe they [providers] also care about the patient, don't they?

### Provider qualitative interviews

We asked providers which guideline recommendations they followed when recommending for or against screening, how they discussed mammography screening with older patients, and about their perceptions around overscreening or overuse of mammography. We identified three major themes: (1) challenges adhering to guideline recommendations for mammography screening among older women, (2) lack of a standardized process or approach to mammography screening for older women, and (3) provider-reported strategies to reduce mammography overuse. The first four interviewees were conducted with primary care providers and the fifth interviewee was an OBGYN.

#### Challenges adhering to guideline recommendations

All providers stated that they followed the guideline recommendations for mammography screening released by their respective professional organizations, mainly the USPSTF and American College of OBGYN. However, providers discussed that providers within their own specialty and/or clinic did not always adhere to these guidelines. As one primary care provider described, “I’ve been here a long time I’ve learned that certain providers are very set in their ways and don’t want intervention.(Interviewee 3)” In addition, providers (2 primary care and 1 OBGYN) described that other providers believe that the benefits of screening outweigh the potential harms of not screening. As one primary care provider reported:

I kind of believe two physicians who basically screen annually until death…So if we have a pretty decent detection test and breast biopsy is a relatively benign procedure, not super morbid, why not just do it? (Interviewee 1)

Providers perceived that their colleague’s decision to continue screening indefinitely may be due to fear of malpractice, past experiences/changing guidelines, and to avoid confusion among older women. According to one primary care provider:

I mean god forbid you tried to convince them to be screened bi-yearly and then they have something on their mammogram and they didn’t get it yearly, like the hospital recommended. You know, like if that ever happened the doctor would be in a terrible position, even though that’s what the guidelines say. (Interviewee 2)

#### Lack of a standardized process or approach to mammography screening

Providers stated that there is no within-system consensus on how to approach screening within this age group. All providers stated that older women receive a letter from outside their clinic reminding women to get an annual mammogram, but it was unclear if the letter came from radiology or the mammography screening clinic. Primary care providers also described how this letter created conflict and confusion around which provider specialty is in charge of mammography screening and made it difficult for them to discuss stopping or reducing screening during an in-person appointment. As one primary care provider described:

I think that this is a really difficult area because even though I feel pretty confident in the guidelines and the data that mammograms should be every two years, our patients get a letter reminding them that they should have their yearly mammogram. I think that that creates a lot of confusion. (Interviewee 2)

In addition, a few providers described how it is challenging to know which women are less likely to benefit from mammography screening and that they did not feel comfortable discussing the pertinence of limited life expectancy for screening with their patients. As one OBGYN provider stated:

That’s really a horrible message to give to people that, “Oh you’re going to die soon so you really don’t need that.” And so if women want to have the imaging then I think that they should and we don’t have the information on older people except to say when people get breast cancer when they’re older it usually grows a little bit slow. (Interviewee 5)

Several providers also reported that an in-person visit was not required for older women to obtain a referral for a mammogram and that older women could call the provider’s office and speak with a nurse who can generate an order or have the referral signed off by another provider who is unfamiliar with the patient’s history. As one primary care provider described:

Now the annual mammogram [letter] that I learn tell people that they are overdue for their mammogram and my patients either will go around me to schedule which they can do or they will alternatively bring me the letters from radiology and say, “This says I’m overdue,” and that’s the whole of the conversation that fairly prompts me to say the same things that I’ve said. (Interviewee 1)

Finally, providers described how mammography overuse is not perceived to be a priority by system leadership, administrators, or other providers. Two providers said that mammography screening is seen as important for revenue generation at the system level. As one primary care provider described:

…then this issue of screening every year, I think unless there’s institutional support for the doctors who want to screen every other year it’s really hard to be put in that position…it’s hard because there’s also a conflict of interest because it’s a moneymaker for Radiology. (Interviewee 2)

#### Provider-reported strategies to reduce mammography overuse

We elicited ideas about potential de-implementation strategies by asking providers how providers and systems could better support older women in being adherent to mammography screening guidelines. Several providers stated that older women and providers could receive educational resources about the harms and limited benefits of mammography screening to help facilitate informed discussions around screening. A couple of providers also suggested utilizing the electronic health record to identify older women for whom reducing the frequency of or stopping mammography screening are recommended (e.g., women with <10-year life expectancy) and to customize system-generated reminder letters based on individual breast cancer risk and health status (i.e., family or personal history of breast cancer; comorbidities). Per two primary care providers:

There’s a couple of pretty decent decisions aids out there for breast cancer screening…Having that integrated in to the EMR in a meaningful way that could be very useful. (Interviewee 1)

I wonder if when you put in the order there was sort of a drop down or a view that you do a patients risk score or a drop-down screening tool…with guidelines that the hospital feels are the guidelines that we should follow. (Interviewee 3)

Finally, a couple of providers emphasized the need for a workgroup comprised of key stakeholders (e.g., primary care providers, OBGYN, radiology, system leadership, administrators) to educate about the harms of mammography overuse; additionally, they could develop a standard process of care around mammography screening that delineates providers’ roles in the referral process and supports a single set of guidelines, to help reduce variation in screening practices and provide recommendations regarding mammography. As one primary care provider summarizes:

What needs to be done in coordination with really a population health perspective on what our given practices are doing about screening more broadly as opposed to us being alone. That will require someone in a leadership level wanting to think about the re-organizing screening tests in some sort of way and think about resources and is there any quality metrics, is there any incentive, how the incentives aligned for that type of a process. So I think that process involves other practices then it makes sense that radiology would be included in that conversation so that we can align similar policies if that was possible. (Interviewee 4)

### Leveraging Norton & Chambers framework to identify multi-level factors and potential de-implementation strategies for mammography overuse

In Table 3, we combined and triangulated quantitative and qualitative data from all sources and organized data according to Norton & Chambers framework [2] to identify factors at the patient, provider, and system-level contributing to mammography overuse. We also grouped and matched potential de-implementation strategies proposed in provider interviews by the level of influence. For example, analysis of all data sources suggested that older women are unaware of the potential harms of overuse and have strong intentions to continue mammography. In response, a primary care provider recommended implementing a decision aid to educate women about the potential harms of screening and recommended that the decision aid be integrated into the electronic health record to help facilitate discussions.

**Table 3:** Multi-level factors perceived to contribute to mammography overuse and potential associated de-implementation strategies

Level	Patient- and provider-identified factors	Provider-identified strategies from interviews
Patient	- Lack of awareness or confusion about overuse - Perception that all healthcare recommended by a provider is necessary - Perceive mammography screening to be important for early detection despite age/health - Perceived need for an annual mammogram	- Develop educational resources for patients, family members, and others involved in mammography screening decisions about the harms of mammography overuse. - Implement or adapt/refine existing decision-aids around mammography screening to older women to facilitate informed discussions around screening.
Provider	- Providers continue to recommend routine mammography screening - Mammography screening practices and guidelines vary across providers within the same clinic and across specialties (e.g. primary care, OB/GYN, radiology) - Challenges identifying older women who are less likely to benefit from mammography screening - Challenges discussing reducing or stopping mammography screening based on age and life expectancy	- Develop educational resources and trainings for providers about the potential harms of mammography overuse. - Leverage the electronic health record to include a decision support system to aid providers and patients in the decision. - Support provider training and feedback regarding communication about mammography overuse and life expectancy.
System/setting	- System-generated reminder letters are in conflict with published guidelines for primary care provider and reinforce mammography screening for older women - System is reimbursed and incentivized for completing mammograms - Mammography overuse is not prioritized or perceived as a problem at the system level - Patients can bypass their providers to get a mammogram and there is no standard referral process	- Patient reminder letters are customized based on individual risk (i.e., family history, personal history). - Modify the electronic health record to include a risk-stratified process for mammography screening. - Convene a system task force to enact consistent mammography screening guidelines across the system - Designate a champion that oversees de-implementation of mammography in older women.

## Discussion

This is one of the first empirical studies to use a mixed-methods approach to understand the factors, processes, and potential strategies at multiple levels that influence mammography overuse in older women. Overall, we found that mammography overuse is not perceived as a priority in our setting and that factors driving overuse are complex and often reinforced through a dynamic interplay of factors across patient, provider, and system levels. Specifically, we found that the characteristics and attributes of older women (e.g., lack of knowledge of the harms, positive attitudes and intentions towards screening), providers (e.g., discomfort discussing screening cessation, fear of malpractice, views on how best to approach screening), and the healthcare system (e.g., variation in how women obtain a mammogram, incentives) all contribute to and reinforce mammography overuse. It is important to note that some of the de-implementation strategies identified by providers span multiple levels, such as education about the harms of overscreening (patient and provider) and integrating educational/training tools into the electronic health record (provider and systems). Moreover, many of the factors contributing to overuse at one level were reinforced or facilitated by factors identified at another level. For instance, system-generated reminder letters reinforced older women’s beliefs and mammography screening behavior and also created challenges for providers discussing the option to reduce or stop screening with their patients. This study also provides valuable insights into potential de-implementation strategies that span multiple levels in our setting; however, more research is needed to understand how these strategies fit the unique needs and context of our patient population, providers, and healthcare system, particularly from an equity perspective.

Findings from this study support that de-implementation likely works differently than implementation processes and is challenging because it works against prevailing practices and beliefs that reinforce the importance of mammography screening [1, 43]. Similar to other studies, older women in our sample hold strong intentions to continue annual mammography screening [29, 44, 45]. Intention has shown to be a strong predictor of mammography screening cessation [46] and should be examined in light of factors that act as barriers and facilitators of mammography screening [47]. In our study, insights from qualitative interviews with older women suggest that screening intention is reinforced by multiple factors, such as having a direct connection to breast cancer (i.e., family history or knowing someone close to their age with breast cancer), receiving a provider recommendation/referral, and a system-generated letter reminder for annual screening. In addition, findings from provider interviews add to the existing literature that primary care providers may be reluctant to discuss reducing mammography screening for older women out of fear of medical malpractice, discomfort discussing sensitive topics, and to avoid confusion from conflicting recommendations around screening [48–50]. We found that this reluctance may be further reinforced by older women’s positive views around screening, a lack of guidance on how to approach mammography screening from a system-level, and financial incentives to continue mammography screening. To this end, greater recognition should be given to the complex interplay between multilevel factors contributing to mammography overuse in older women.

It is well documented that system-level factors or characteristics including practice size, type, resources, staffing, organizational culture, and cost influence cancer screening but less attention has focused on the care delivery process [1, 47]. Our findings suggest that there is variation in mammography screening practices (i.e., referrals, scheduling appointment, guideline implementation) across providers and increased opportunities for older women to obtain a mammogram. While some practice variation may be justified when based on older women’s health and preferences, as our findings support, variation resulting from conflicting guideline recommendations [51], lack of care continuity [52], lack of communication [53], or teamwork [54] between providers can contribute to healthcare overuse [1, 7, 47]. De-implementation science frameworks acknowledge the need to examine practice variation and the overall care delivery process early on in the process of identifying and prioritizing low-value practices [55]. However, few acknowledge practice variation or the larger care delivery process as a factor or determinant contributing to overuse, despite studies linking the care delivery process to overuse [47]. Particularly for long-standing and embedded practices, such as mammography screening, more empirical work in this area is needed to better understand the role of the care delivery process early on in the de-implementation process, as well as a determinant of overuse to inform the development of system-level strategies to reduce variation.

A large proportion of our ethnically diverse sample believed it was important to plan to have a yearly mammogram (87.2% survey, 88.9% interview), and all received a mammogram in the last 12 months. These findings support the broader notion that racial/ethnic minority populations are also susceptible to overuse that is perpetuated by providers and the operations of the system [36, 56, 57]. To date, few studies on de-implementation capture the perspectives and experiences of ethnically diverse populations or of older women more broadly [29]. In fact, a recent scoping review of de-implementation science theories, models, and frameworks found only two that account for the role of patients [57]. Similar to prior studies, findings from qualitative interviews with older women found that the majority had not heard of or experienced excessive or unnecessary care and struggled to understand how receiving a mammogram could lead to harm [58, 59]. This perception combined with societal norms in the USA related to successful population-level efforts to address mammography underuse and limited training or preparation of providers to address overuse, may further impede de-implementation [6, 60, 61]. These findings support the need for future research to understand the magnitude of and perspectives towards mammography overuse across diverse settings and populations, with the ultimate goal of helping to advance the science of achieving health equity through de-implementation.

There is growing recognition that strategies for de-implementation likely differ from strategies used for implementation [57], but there is little evidence to indicate what these specific strategies may be and how to best develop them. This study provides a glimpse into potential strategies specific to de-implementation; further research is needed to understand what strategies providers perceive to be feasible and acceptable and to develop/test the effectiveness of such strategies. As seen in our study, strategies including stakeholder engagement and leadership buy-in are likely effective for both implementation and de-implementation [1]. In addition, strategies that target habit formation and disruption by changing environmental cues (e.g., leveraging the electronic health record to identify women most likely to benefit from screening, tailoring system-generated letters accordingly) and behavior (e.g., having the primary care provider review and sign off on all mammography referrals for their patients) may help to reduce variation in the mammography screening processes [62, 63]. Our findings also support the need for strategies that target professional biases to foster de-implementation, such as ensuring that system-level workgroups include experts from different provider specialties, emphasizing evidence and guidelines over individual clinical judgement, and encouraging providers to consider how their experiences bias their interpretations of clinical evidence [64]. Importantly, de-implementation strategies should be designed and refined with health equity in mind by considering racially and ethnically diverse older women’s experiences and perceptions of overuse and should reflect their cultural or communication preferences, or specific structural barriers faced [36, 56].

Our study has several important strengths. The use of a mixed-methods design facilitated a deeper understanding of factors and potential mechanisms influencing mammography overuse and inform potential de-implementation strategies. We also present findings from multiple perspectives and sources including from predominately older, ethnically diverse women who are underrepresented in research to date. In addition, we were able to clinically identify women who were active screeners using the electronic health record rather than through self-report. However, important limitations should be considered when interpreting results. First, the survey and interview design were not informed by theories, models, or frameworks specific to de-implementation, but instead sought to broadly understand multi-level factors contributing to mammography overuse in our setting. However, our findings provide insight into potential gaps in current conceptualizations of de-implementation of mammography overuse and we utilized the *Continuum of Factors Influencing De-implementation Process* [2] framework to help organize our results, which provides a solid foundation for future research in this area. Second, findings from provider interviews may not represent the entire spectrum of experiences or perspectives around mammography overuse due to the small sample size. Provider interviews were also limited to primary care and OBGYN providers and did not include the perspectives of other specialties, such as radiology, or system leadership who also contribute to mammography overuse. Despite small sample sizes, all providers corroborated findings from surveys and interviews with older women and were purposefully sampled to serve as key informants representing the broader views of providers involved with mammography screening among older women. A notable strength of our study is the inclusion of older, ethnically diverse women who may be susceptible to underuse and overuse of mammography screening. However, our findings do include perspectives from women less than 75 years of age for which mammography screening is appropriate. Finally, all participants were recruited from a single healthcare system, limiting our ability to generalize to other settings. Yet, these findings may serve as a starting point for other healthcare systems by providing perspectives of what strategies may be preferred and acceptable by providers and what types of multilevel factors may hinder de-implementation efforts. In addition, the overall synthesis of results provides important information to help advance the science of de-implementation by informing context-specific strategies and hypothesis-generating directions for future research.

## Conclusion

This study aligns with calls to advance the science of de-implementation and includes the experiences and perspectives of older women underrepresented in research to date. Our study emphasizes the need for further refinement and empirical testing of de-implementation theories, models, and frameworks that incorporate a strong patient-level component and considers the complex interplay between multi-level factors within the larger process of care delivery. In addition, findings from provider interviews suggest potential de-implementation strategies to reduce mammography overuse that are context-specific, spanning multiple levels, and informed by health behavior and organizational theory, as well as, theories of habit formation and disruption. Finally, these findings point to the need for more robust mixed-methods studies aimed at understanding the magnitude of mammography overuse and multi-level factors influencing mammography screening across diverse populations and settings to aid in the development and testing of strategies tailored to the context and needs of the population.

## Supplementary Information

**Additional file 1: Supplement.** Characteristics of survey and semi-structured interview participants.

## Data Availability

All data generated or analyzed during this study are available from the corresponding author on reasonable request.
