# Supplementary material for: A mixed-methods study of multi-level factors influencing mammography overuse among an older ethnically diverse screening population: implications for de-implementation
Source: Implement Sci Commun. 2021 Sep 26;2:110. doi: 10.1186/s43058-021-00217-7 (PMC8474751; doi:10.1186/s43058-021-00217-7)
Supplement: Supplementary file 1 — Additional file 1: Supplement. Characteristics of survey and semi-structured interview participants. [file 43058_2021_217_MOESM1_ESM.docx]

| **Supplement.** Characteristics of survey and semi-structured interview participants. | | |
| --- | --- | --- |
|  | **Overall (n = 52)** | **Completed qualitative interview (n = 19)** |
| **Age** | Mean (SD): 74.6 (3.76)  Range: 70 - 89 | Mean (SD): 74.9 (3.78)  Range: 71 - 83 |
| **Years in the US** | Mean (SD): 41.6 (12.8)  Range:14 - 61 | Mean (SD): 44.8 (12.5)  Range: 20 - 61 |
|  | **n(%)** | **n(%)** |
| **Race**  Hispanic  Non-Hispanic white  Non-Hispanic black  Non- Hispanic Other | 38 (73.08)  11 (21.15)  1 (1.92)  2 (3.85) | 12 (63.16)  6 (31.58)  1 (5.26)  -- |
| **Nativity**  US-born  Foreign – born | 10 (19.23)  42 (80.77) | 6 (31.58)  13 (68.42) |
| **Country of Origin**  US  Dominican Republic  Cuba  Mexico  Ecuador  Puerto Rico  Russia  Other country (Belize, Colombia, Germany, Philippines) | 10 (19.23)  32 (61.54)  1 (1.92)  1 (1.92)  2 (3.85)  --  1 (1.92)  5 (9.62) | 6 (31.58)  7 (36.84)  1 (5.26)  1 (5.26)  2 (10.53)  --  --  2 (10.53) |
| **Interview Language**  Spanish  English | 38 (73.08)  14 (26.92) | 12 (63.16)  7 (36.84) |
| **Education**  Less than high school  High school or GED  Some college  Bacherlors or advanced degree | 23 (44.23)  8 (15.38)  8 (15.38)  10 (19.23) | 7 (36.84)  3 (15.79)  4 (21.05)  5 (26.32) |
| **Health Literacy**  Adequate  Marginal  Low | 17 (32.69)  15(28.85)  15(28.85) | 7 (36.84)  7 (36.84)  5 (26.32) |
| **Marital Status**  Married  Single, never married  Divorced or separated  Window | 14 (26.92)  13 (25.0)  12 (23.08)  9 (17.31) | 4 (21.05)  5 (26.32)  6 (31.58)  4 (21.05) |
| **Number of Chronic Conditions**  1-2  3-4  5+ | 9 (17.31)  23 (44.23)  20 (38.46) | 2 (10.53)  9 (47.37)  8 ( 42.11) |
| **Self-reported Health**  Poor  Fair  Good  Very good  Excellent | 6 (11.54)  20 (38.46)  11 (21.15)  11 (21.15)  3 (5.77) | 3 (15.79)  7 (36.84)  3 (15.79)  6 (31.58)  -- |
| **Family History of Breast Cancer (1^st^ degree female relatives)**  No  Yes | 45 (86.54)  7 (13.46) | 16 (84.21)  3 (15.79) |
| **History of Diagnostic Follow-Up (Q13)**  No  Yes | 29 (55.77)  23 (44.23) | 11 (57.89)  8 (42.11) |
